# Valproate and Short-Chain Fatty Acids Activate Transcription of the Human Vitamin D Receptor Gene through a Proximal GC-Rich DNA Region Containing Two Putative Sp1 Binding Sites

**DOI:** 10.3390/nu14132673

**Published:** 2022-06-28

**Authors:** Marta Moreno-Torres, Carla Guzmán, Petar D. Petrov, Ramiro Jover

**Affiliations:** 1 Unidad de Hepatología Experimental, Instituto de Investigación Sanitaria Hospital La Fe, 46026 Valencia, Spain; guzman_capri@gva.es (C.G.); petar.petrov@ciberehd.org (P.D.P.); 2 CIBEREHD, Instituto de Salud Carlos III, 28029 Madrid, Spain; 3 Departamento de Bioquímica y Biología Molecular, Facultad de Medicina, Universidad de Valencia, 46010 Valencia, Spain

**Keywords:** VDR induction, human VDR promoter, valproic acid, SCFA, Sp1.

## Abstract

The vitamin D receptor (VDR) mediates 1,25-dihydroxyvitamin D3 pleiotropic biological actions through transcription regulation of target genes. The expression levels of this ligand-activated nuclear receptor are regulated by multiple mechanisms both at transcriptional and post-transcriptional levels. Vitamin D3 is the natural VDR activator, but other molecules and signaling pathways have also been reported to regulate VDR expression and activity. In this study, we identify valproic acid (VPA) and natural short-chain fatty acids (SCFAs) as novel transcriptional activators of the human VDR (hVDR) gene. We further report a comprehensive characterization of VPA/SCFA-responsive elements in the 5′ regulatory region of the hVDR gene. Two alternative promoter DNA regions (of 2.4 and 3.8 kb), as well as subsequent deletion fragments, were cloned in pGL4-LUC reporter vector. Transfection of these constructs in HepG2 and human Upcyte hepatocytes followed by reporter assays demonstrated that a region of 107 bp (from −107 to −1) upstream of the transcription start site in exon 1a is responsible for most of the increase in transcriptional activity in response to VPA/SCFAs. This short DNA region is GC-rich, does not contain an apparent TATA box, and includes two bona fide binding sites for the transcription factor Sp1. Our results substantiate the hypothesis that VPA and SCFAs facilitate the activity of Sp1 on novel Sp1 responsive elements in the hVDR gene, thus promoting VDR upregulation and signaling. Elevated hepatic VDR levels have been associated with liver steatosis and, therefore, our results may have clinical relevance in epileptic pediatric patients on VPA therapy. Our results could also be suggestive of VDR upregulation by SCFAs produced by gut microbiota.

## 1. Introduction

Vitamin D receptor (VDR) is a member of the nuclear receptor superfamily of ligand-activated transcription factors, which is expressed in multiple normal and transformed cell types. The promoter regions of many nuclear receptor genes have been characterized. They resemble housekeeping genes, and many are embedded in GpC islands [1,2]. Another common feature is the absence of a TATA box as well as the existence of multiple transcription start sites. In addition, mRNA transcripts can be produced from alternative promoters [3,4,5] and transcripts can be differentially spliced to form unique mRNA species to create functionally distinct receptor isoforms [6,7].

**Figure 1:**
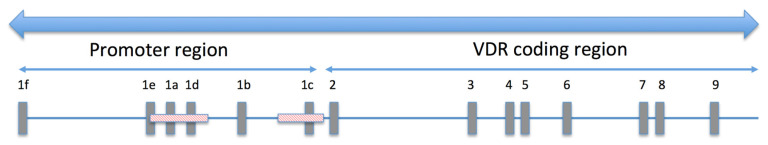
Schematic representation of hVDR (human Vitamin D Receptor) gene locus. The human VDR gene consists of 14 exons, six of which are different variants of exon 1, participating in the process of alternative splicing. Exons 2–9 are common to all known VDR protein isoforms. Regions in red indicate the area of two previously described putative promoter regions [10,11].

Specifically, VDR was discovered as a protein activated by 1,25-dihydroxyvitamin D3 (1,25(OH)_2_D3, calcitriol or VitD3), the hormonal form of cholecalciferol [8]. The gene of approximately 100 kb includes 14 exons, and it is divided into two main regions (Figure 1). The first exon, which is located in the promotor region, presents six variants (a−f) relevant for VDR alternative splicing. Exons 2–9 are located in the coding region and are common for all VDR transcripts [9].

VDR is a member of the superfamily of nuclear steroid/thyroid hormone receptors that localizes in the cytoplasm and nucleus of the cells. Following ligand-binding, the VDR forms a heterodimer with retinoid X receptor (RXR) to regulate gene expression via binding to vitamin-D-responsive elements (VDRE) in regulatory regions of target genes. The VitD3/VDR signaling pathway controls calcium and phosphorous homeostasis, immune response, hormonal systems, and cell growth [12]. The growth regulatory effects involve proliferation inhibition, differentiation induction, and apoptosis activation [13,14].

VDR abundance is controlled by transcriptional and post-transcriptional (mRNA stability) regulations, post-translational modifications, and ligand-induced receptor stabilization [9]. At the post-translational level it has been demonstrated that the secondary bile acid lithocholic acid (LCA) is another ligand for VDR activation [15].

Regarding VDR gene transcription, several mechanisms are involved. First, homologous upregulation of VDR mRNA by VitD3, the natural ligand for VDR, has been demonstrated both in vitro and in vivo, in a tissue-dependent manner [16,17]. In addition, VDR gene expression is upregulated in NIH-3T3 mouse fibroblasts through activation of the protein kinase A pathway [18] and downregulated through activation of the protein kinase C pathway [19]. In addition, other growth factors and cytokines are also known to regulate VDR gene expression. Estrogens [20], thyroid hormone [21], glucocorticoids [22], and retinoic acid [23,24] are likewise able to alter VDR mRNA levels in what appears to be tissue-specific patterns of expression. Furthermore, both the cell cycle [25] and differentiation state [26] influence the levels of VDR mRNA.

In previous studies we identified VDR as a pro-lipogenic nuclear receptor, that upon activation, triggers a coordinated gene response causing alterations in glycerolipid and phospholipid metabolism and leading to triglyceride accumulation in hepatocytes [27,28]. We and others have also demonstrated that valproic acid (VPA) is one of the most potent pro-lipogenic drugs, and causes liver steatosis in patients and in in vitro models [29,30]. We, therefore, hypothesized that one potential mechanism of VPA-induced steatosis could be through induction of the pro-lipogenic VDR gene.

VPA has been the first-line, most-frequently prescribed, anti-epileptic drug in children for the past fifty years [31] and is also commonly used today to treat bipolar disorder (maniac depressive illness). VPA is a branched short-chain fatty acid and consequently it shares chemical properties with natural SCFAs such as butyrate. VPA, similarly to butyrate, has recognized histone deacetylase 1 (HDAC1) inhibitory capacity (IC50 = 0.4 mM) [32], and therefore it is expected to have a significant impact on gene expression. Indeed, VPA alters the expression of more than 1900 genes in mouse liver [33]. Recent evidence also demonstrates that VPA increased expression of CYP24A1, a VDR target gene that catabolizes VitD3 at physiological concentrations [34]. However, the effect of VPA/SCFAs on VDR expression regulation has not yet been investigated.

Here we identify a novel role of VPA/SCFAs in the induction of VDR expression in human hepatocytes and investigate the transcriptional activation of VDR by VPA/SCFAs through specific promoter DNA elements. For that purpose, two 5′ flanking regions of the VDR gene, previously described as promoter regions, were cloned upstream of the luciferase-coding gene to check functional activity in two different human hepatic cell models. After deletion and segmentation, a short promoter region of 107 bp, which includes two putative Sp1 binding sites, was identified as the principal VPA/SCFA response region in the hVDR gene.

## 2. Materials and Methods

### 2.1. Cell Culture and Treatment of HepG2 Cells

HepG2 cells were routinely grown in culture grade flasks at 37 °C under a humidified atmosphere 5% CO_2_/95% air in Ham’s F-12/Leibovitz L-15 (1:1, *v*/*v*) supplemented with 7% fetal bovine serum (Capricorn Scientific GmbH, Ebsdorfergrund, Germany), 50 U/mL penicillin (Gibco, Waltham, MA, USA), and 50 μg/mL streptomycin (Gibco). Cells were used or passaged at 70–80% confluence. For subculturing purposes, cells were detached with 0.25% trypsin/0.02% EDTA in PBS at 37 °C.

Human Upcyte^®^ hepatocytes (Upcyte^®^ Technologies GmbH, Hamburg, Germany) are cells derived from primary human hepatocytes by a novel technology that forces cells to a limited proliferation, without immortalization, while maintaining specific liver functions. Unlike HepG2 cells, they express characteristic hepatic genes [5] and preserve a phenotype close to that of primary cultured human hepatocytes [35,36]. Upcyte hepatocytes were cultured essentially as previously described [36].

Cells were treated with the appropriate compound or with an equal volume of vehicle (PBS) for 24 h. Valproate (Cat. no. P4543), butyrate (Cat. no. B5887), octanoate (Cat. no. C5038), hexanoate (Cat. no. C4026), propionate (Cat. no. P1880), and acetate (Cat. no. S2889) were purchase from Sigma-Aldrich (Taufkirchen, Germany).

### 2.2. RNA Extraction and Expression Analysis

Total RNA was purified from cultured cells by using a mixed TriZol–column-based protocol. More specifically, following lysis with TriZol and phase separation with chloroform, the aqueous phase was mixed with an equal volume of buffer and processed according to the protocol of RNAeasy kit (Qiagen). The integrity of total RNA was assessed by running 500 ng of total RNA on 1% agarose gel and by a microcapillary electrophoresis (2100 Bioanalyzer, Agilent Technologies, CA, USA), whereas purity was assessed by the A260/A280 ratio obtained after measurement with NanoDrop1000.

Gene expression analysis by RT-qPCR were performed essentially as already described [37]. PBGD (porphobilinogen deaminase) was used as reference gene, as its expressions were stable in the different conditions. Primers used are listed in Table 1.

### 2.3. PCR Amplification of Two Promoter Regions of the hVDR Gene

A sequence of 3836 bp encompassing exons 1e, 1a, and 1d (fragment between −1899 and +1937 around the transcription start site of exon 1a) and a sequence of 2440 bp upstream and within exon 1c (fragment between −1253 and +1187 of the transcription start site of exon 1c) of the hVDR gene previously described as putative promoters [10,11] were amplified by high-fidelity PCR. These PCRs were performed by using the enzyme Phusion™ High-Fidelity DNA Polymerase (2 U/µL) (ThermoScientific, MA, USA). For these two sequences genomic DNA (Affimetrix, CA, USA) was used as a template. A set of specific primers (VDR-UP3094-KpnI, VDR-DN6813-HindIII, VDR-UP25869-KpnI, and VDR-DN28360-HindIII) for these regions was designed and synthesized (Table 1).

### 2.4. Construction of Luciferase Reporter Plasmids and Transient Transfections

Chimeric luciferase reporter constructs with the two fragments of 3836 bp (from −1899 to +1937 around exon 1a transcription start site) and 2440 bp (from −1253 to +1187 around exon 1c transcription start site) of the flanking region of the human VDR gene were obtained by cloning the PCR products (digested with KpnI-HindIII) into the promoterless pGL4.10 vector (Promega Corp., Madison, WI, USA) previously digested with the same enzymes, which contains the firefly luciferase reporter gene. Lack of mutations in the cloned sequence was assessed by Sanger DNA sequencing and contig assembly.

pGL4.10-VDR-3836 was digested with enzymes KpnI-EcoRV, to get a fragment of 1307 bp (−1899 to −592), and EcoRV-HindIII to get a fragment of 2528 bp (−591 to +1937) which were subcloned into pGL4.10.

The sequences of 1438 bp (+499 to +1937), 1968 bp (−98 to +1870), and 489 bp (−591 to −102) were obtained by digesting the plasmid pGL4-VDR-2528 with ApaI, SacI, and KpnI-SacI, respectively, and subsequently subcloned in pGL4.10 digested with same enzymes.

The sequence of 597 bp (−98 to +499) was obtained by digesting the plasmid pGL4-VDR-3836 with SacI-ApaI, and the resulting fragment was ligated into pGL4.10 digested with same enzymes.

Sequences corresponding to 245 bp (−97 to +148) and to 332 bp (+167 to +499), were PCR cloned from pGL4-597-VDR with specific primers (508-VDR:161U26 and 508-VDR:421L27, 508-VDR:433U26, and 508-VDR:672L27, respectively). Amplicons were digested with KpnI and XhoI and the resulting fragment was ligated into pGL4.10 digested with same enzymes.

Sequences corresponding to 107 bp (−107 to −1), 96 bp (−7 to +89), and 60 bp (+88 to +148) were PCR cloned from pGL4-332-VDR plasmid as previously described with specific primers (VDR-287_1-Sac-UP and VDR-287_1-Hind-DN, VDR-287_2-Sac-UP and VDR-287_2-Hind-DN, VDR-287_3-Sac-UP, and VDR-287_3-Hind-DN, respectively). Amplicons were digested with SacI and HindIII and the resulting fragment was ligated into pGL4.10 digested with same enzymes.

Transient transfections with VDR-reporter plasmids were performed in HepG2 and Upcyte cells seeded in 24-well plates at a density of 1.4 × 10^5^ cells per well. Cells were incubated at 37 °C overnight, and 1 h before transfection, medium was replaced with fresh antibiotic-free medium. Prior to transfection 200 ng of Renilla-coding pRL-CMV vector (for normalization) and equimolar amounts of either pGL4.10 empty vector or VDR-promoter constructs were mixed in Opti-MEM media, followed by the addition of the reagent X-tremeGENE HP DNA Transfection Reagent (Roche Applied Science, Penzberg, Germany) in a 1:1 DNA:lipid ratio. The mixture was incubated for complex formation at room temperature for 30 min. DNA:lipid complexes were added dropwise to the cells. Next day, medium was changed, and VPA/SCFAs were added to the cells for 24 h to activate reporter constructs. Cell lysates were prepared by applying PLB buffer (Promega). Lysates were cleared by centrifugation at 10,000× *g* for 1 min. Next, 10 µL of each lysate were pipetted in a white microplate, followed by addition of 30 µL of LAR II reagent, and the luminescence produced by firefly luciferase was recorded. Finally, 30 µL of Stop and Glo reagent (Promega) was added to quench the firefly signal and to detect the luminescence produced by the Renilla luciferase. Each experiment was performed in duplicate and replicated between three and eight times.

### 2.5. Statistics

The luciferase assay data were statistically analyzed by *t*-test or ANOVA as appropriate, with Graph Pad Instat Software (San Diego, CA, USA). Means were considered significantly different if *p*-values < 0.05 were obtained. Quantitative variables were expressed as mean ± standard error of the mean (SEM), but individual values for each replicate were also depicted.

## 3. Results

### 3.1. VAP and SCFA (Butyrate) Induce VDR mRNA in HepG2 Cells and Human Hepatocytes

In a previous study, we demonstrated that active VitD3/VDR signaling triggers a coordinated gene response leading to triglyceride synthesis and to perturbations in lipid metabolism, specifically in glycerolipids and phospholipids [27]. The above-mentioned evidence led us to investigate whether natural SCFAs and drug analogues (VPA) were able to influence VDR expression.

**Figure 2:**
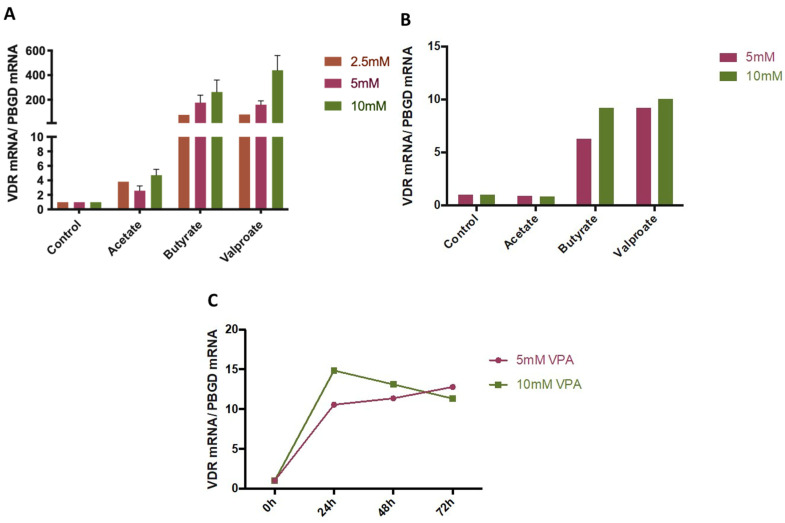
hVDR (human Vitamin D Receptor) mRNA expression levels after treatment with VPA(valproic acid)/SCFAs (short chain fatty acids). HepG2 (**A**) and Upcyte hepatocytes (**B**) were treated with 2.5, 5, and 10 mM of acetate, butyrate, and VPA for 24 h. Extended incubation times (48 h and 72 h) were investigated in Upcyte hepatocytes (**C**). Results are expressed as mean ± SEM from 2–3 independent experiments (**A**) or as average of duplicates from a single experiment (B) and are represented as fold-change relative to vehicle (control) (**A**,**B**) or to time 0 h (**C**).

Cells were treated with sub-cytotoxic concentrations (2.5–10 mM) of SCFAs (butyrate and acetate) or VPA and VDR mRNA levels analyzed by RT-qPCR. No significant effect was observed for acetate, whereas butyrate and VPA dose-dependently induced VDR expression both in HepG2 and hepatocytes (Figure 2A,B). A time-course experiment demonstrated that maximal induction of VDR mRNA was reached by 24 h, without further increase at later time points (Figure 2C).

### 3.2. Functional Analysis of the hVDR Promoter

Previous studies [10,11] delimited two different DNA sequences in the hVDR gene with potential promoter activity (Region 1: 3836 bp, Region 2: 2440 bp) (Figure 1). These two regions were PCR-amplified and cloned into the promoterless firefly luciferase plasmid pGL4.10, and transiently transfected into HepG2 cells and human hepatocytes to evaluate transcriptional activity by reporter assay.

**Figure 3:**
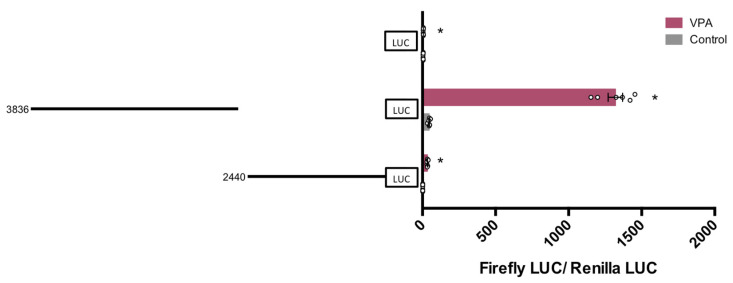
Response of two putative hVDR (human Vitamin D Receptor) gene promoter regions of 3836 bp and 2440 bp to VPA (valproic acid). HepG2 cells were transfected with the pGL4-VDR-3836 (sequence from −1899 to +1937 around exon 1a transcription start site), pGL4-VDR-2440 (sequence from −1253 to +1187 around exon 1c transcription start site), or pGL4 empty vector and treated with 5 mM VPA, or vehicle. Firefly luciferase activity was normalized with Renilla luciferase activity (relative light units, RLUs). Results are expressed as mean ± SEM from six independent experiments. * *p*-value < 0.05, *t*-test.

As shown in Figure 3, the activity of the pGL4.10-VDR-3836 construct had a noticeable basal activity level and was strongly induced by VPA (~29 fold-increase), while much lower basal and induced levels were observed in cells with pGL4.10-VDR-2440. Thus, results demonstrated that the sequence of 3836 bp includes the principal DNA response elements mediating the transcriptional activation of hVDR by VPA.

To precisely delineate the DNA sequence responsible for hVDR activation by VPA, further deletions were obtained and cloned. First, the 3836 bp sequence was fragmented into two regions of 1307 bp and 2528 bp.

**Figure 4:**
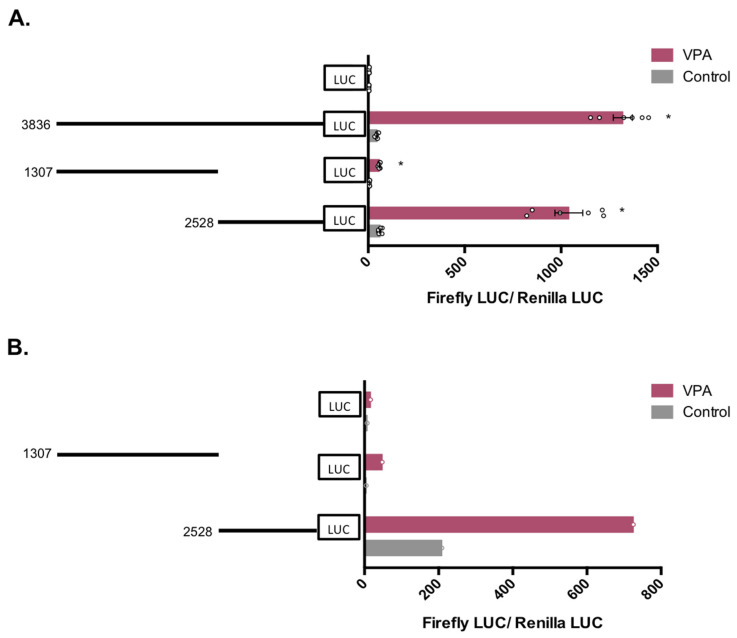
Response of three hVDR (human Vitamin D Receptor) gene promoter regions of 3836 bp, 1307 bp, and 2528 bp to VPA (valproic acid). HepG2 cells (**A**) or human hepatocytes (**B**) were transfected with the pGL4-VDR-3836 (−1899 to +1937), pGL4-VDR-1307 (−1899 to −592), pGL4-VDR-2528 (−591 to +1937), or pGL4 empty vector and treated with 5 mM VPA, or vehicle. Firefly luciferase activity was normalized with Renilla luciferase activity (relative light units, RLUs). Results are expressed as mean ± SEM from six independent experiments. * *p*-value < 0.05, *t*-test (**A**). Single experiment values are shown for Upcyte hepatocytes (**B**).

As shown in Figure 4, transfection of these fragments in HepG2 cells revealed that the downstream 2528 bp sequence retained almost all activity observed in the 3836 bp sequence, exhibiting an average 17-fold increase in response to VPA, whereas the upstream 1307 bp sequence, although still inducing activity, had only an 8-fold increase (Figure 4A). Similar results were obtained in hepatocytes, where the construct pGL4.10-VDR-2528 displayed the highest promoter activity upon VPA treatment (Figure 4B).

**Figure 5:**
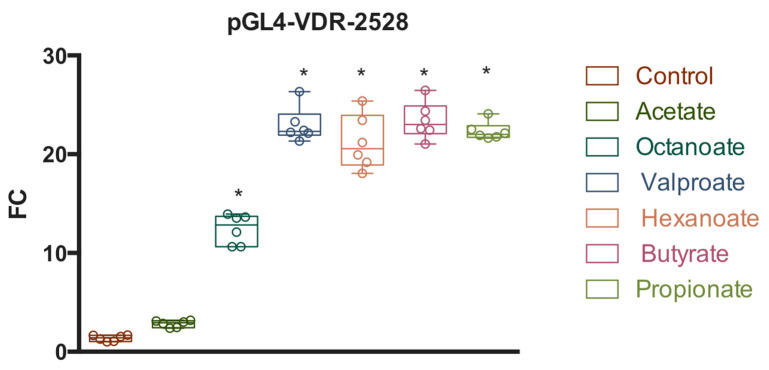
Response of a hVDR (human Vitamin D Receptor) gene promoter region of 2528 bp to SCFAs (short chain fatty acids). HepG2 cells were transfected with the pGL4-VDR-2528 (−591 to +1937) construct or pGL4 empty vector and treated with vehicle (control), acetate (5 mM), octanoate (5 mM), VPA (5 mM), hexanoate (5 mM), butyrate (5 mM), and propionate (5 mM). After 24 h, firefly luciferase activity was measured and normalized with Renilla luciferase activity (relative light units, RLUs). Results are expressed as mean ± SEM from six independent experiments. * *p*-value < 0.05, one-way ANOVA with Tukey’s multiple comparisons test.

Subsequently, we determined whether natural SCFAs also modulate reporter gene activity of the construct pGL.4-VDR-2528. Similar reporter assays were conducted in HepG2 cells treated with acetate, octanoate, hexanoate, butyrate, propionate, and VPA. As shown in Figure 5, all SCFAs, except acetate, upregulated the promoter activity above the basal levels seen in vehicle-treated control cells expressing the same plasmid. Octanoate increased promoter activity by approximately 12-fold, whereas VPA, hexanoate, butyrate and propionate triggered increases of approximately 22-fold, suggesting that the 2528 bp sequence strongly responds to regulation by SCFAs.

To further delimitate the VDR gene sequence responsive to VPA/SCFAs, the fragment of 2528 bp was subsequently divided into shorter regions: 1968 bp (−98 to +1870), 1438 bp (+499 to +1937), and 489 bp (−591 to −102).

**Figure 6:**
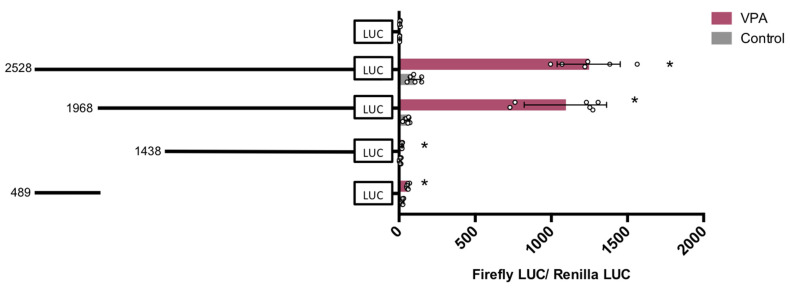
Response of four hVDR (human Vitamin D Receptor) gene promoter regions of 2528 bp, 1968 bp, 1438 bp, and 489 bp to VPA (valproic acid). HepG2 cells were transfected with the pGL4-VDR-2528 (−591 to +1937), 1968 (−98 to +1870), 1438 (+499 to +1937), and 489 (−591 to −102) plasmids or pGL4 empty vector and treated with 5 mM VPA, or vehicle as control. Firefly luciferase activity was normalized with Renilla luciferase activity (relative light units, RLUs). Results are expressed as mean ± SEM from six independent experiments. * *p*-value < 0.05, *t*-test.

Promoter functionality of the new constructs was again evaluated with luciferase reporter assays (Figure 6). The 1968 bp construct kept promoter activity upon VPA treatment to a similar extent as the original longer 2528 bp construct, with a fold increase of 20 and 12 over control treated cells expressing the same vector, respectively. However, both 489 bp and 1438 bp constructs lost almost all reporter activity, which suggests that the region included in 1968 bp and not in 1438 bp sequence is the sequence responsible for promoter activation by VPA.

**Figure 7:**
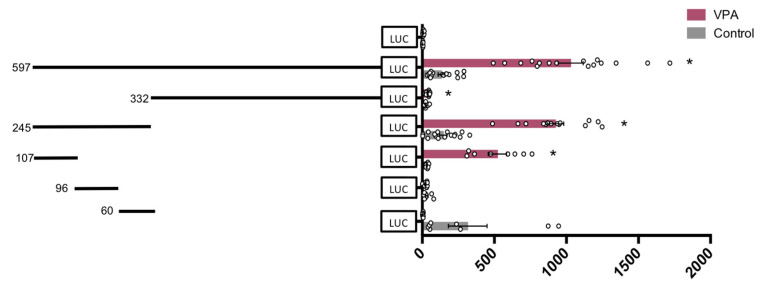
Response of six hVDR (human Vitamin D Receptor) gene promoter regions of 597 bp, 332 bp, 245 bp, 107 bp, 96 bp, and 60 bp to VPA (valproic acid). HepG2 cells were transfected with the pGL4-VDR-597 (−98 to +499), 332 (+167 to +499), 245 (−97 to +148), 107 (−107 to −1), 96 (−7 to +89), and 60 (+89 to +148) constructs or pGL4 empty vector and treated with 5 mM valproate, or vehicle as control. Firefly luciferase activity was normalized with Renilla luciferase activity (relative light units, RLUs). Results are expressed as mean ± SEM from *n* ≥ 8 independent experiments. * *p*-value < 0.05, *t*-test.

To gain further insight into the sequence of interest (between −98 and +499), pGL4-VDR-3836 was digested with ApaI and SacI to obtain the sequence of 597 bp (−98 to +499) which was cloned and two subsequent fragment regions of 245 bp (−97 to +148) and 332 bp (+167 to +499) were amplified by PCR from pGL4.10-3836 and subcloned into pGL4.10. Results shown in Figure 7 evidence that the region of 245 bp is the only one responsible for VPA-mediated induction of VDR promoter activity. We split this region further into three sections of 107 bp (−107 to −1), 96 bp (−7 to +89), and 60 bp (+88 to +148) that were amplified by PCR from pGL4.10-245 and subcloned into pGL4.10 with enzymes SacI y HindIII.

**Figure 8:**
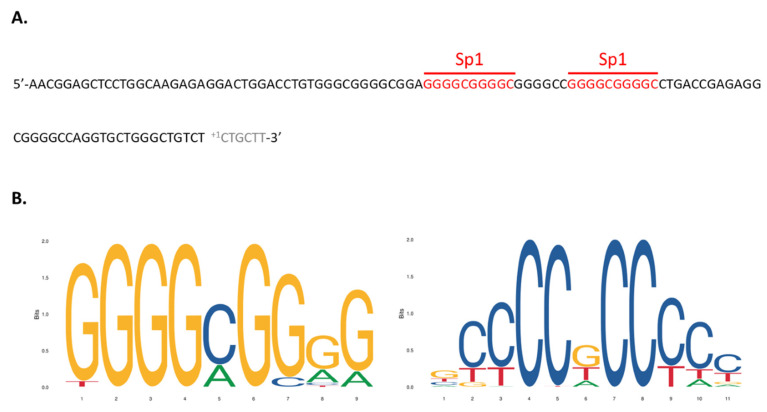
VPA(valproic acid)/SCFA(short chain fatty acids)-responsive sequence in the promoter of the hVDR (human Vitamin D Receptor) gene. (**A**) Two Sp1 binding sites have been identified in the 107 bp sequence. (**B**) Sp1 consensus binding sequences from JASPAR database.

Results in Figure 7 evidence that the upstream 107 bp sequence is the main DNA element that mediated the induction of VDR by VPA. This novel promoter region of the hVDR lies in a GpC island, does not contain a TATA box, and contains two Sp1 binding sites (Figure 8). Sp1 is known to interact both with a number of cellular and viral promoters as well as other transcription factors. Our results demonstrate that the promoter region has a substantial capacity to promote transcription of a reporter gene in response to VPA and to natural SCFAs. Our results substantiate the hypothesis that Sp1 transcription factors may play a relevant role in this activation of the hVDR gene.

## 4. Discussion

To our knowledge this is the first study showing that VPA and natural SCFAs are able to transcriptionally upregulate the hVDR gene. To gain insight into this novel mechanism of hVDR regulation, we cloned two 5’ upstream regions previously described as putative promoters [10,11] and evaluated their activity in luciferase reporter assays. Following subsequent fragmentation into shorter sequences and the assessment of their functionality, we finally identified a 107 bp proximal promoter sequence (just upstream of nt +1 in exon 1a) acting as the responsive region in the hVDR gene. The sequence was in silico analyzed for putative transcription factor binding sites using tools such as Match and databases such as Transfact [38]. Interestingly, no TATA box but two Sp1 sites were found in this region. Sp1 belongs to the family of zinc finger transcription factors, it can bind to GC-rich motifs in the promoter and regulate multiple house-keeping and growth related genes [39,40].

**Figure 9:**
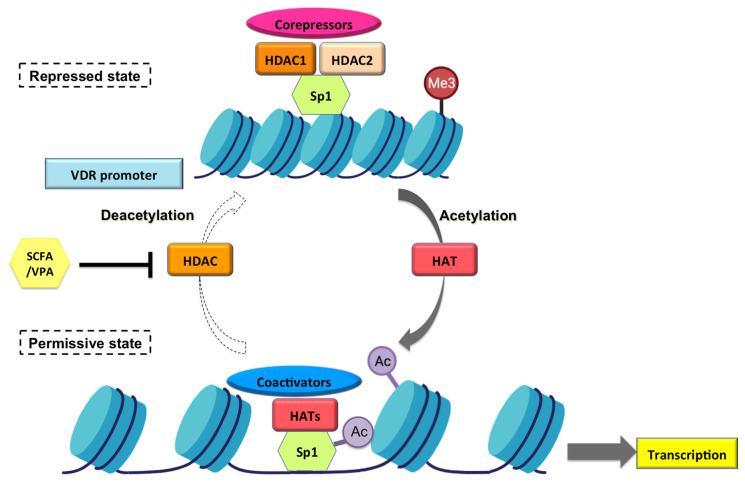
Schematic hypothetical model of VPA (valproic acid)-mediated induction of VDR (Vitamin D Receptor) gene transcription and expression via inhibition of HDACs (histone deacetylases) and chromatin remodeling. The schematic model shows that under untreated conditions class I HDACs (HDAC-1 and -2) interact with Sp1 and form a correpressor complex mantaining histones hypoacetylated.Under these conditions, chromatin is in its non-permissive or repressed state and Sp1 transcription factor has low affinity for the promoter. After treatment with VPA, its HDACi activity inhibits HDACs, which do not compete with HATs. Upon acetylation of histones (and Sp1), chromatin opens and Sp1 occupies the VDR promoter leading to VDR gene transcription. Figure adapted from [43].

Although the mechanisms by which VPA/SCFAs modulate Sp1 and upregulate VDR transcription has not been addressed in this study, we speculate on several hypothesis based on previous knowledge of VPA and SCFA modes of action (Figure 9). The recruitment of histone acetyltransferases (HATs) and histone deacetylases (HDACs) is a key process in the dynamic regulation of many genes involved in several processes such as proliferation and differentiation. Acetylation of the NH_2_-terminal tails of core histones is central in the specification of a “histone code” that influences the expression of target genes. Recruitment of histone acetyltransferases by transcription factor complexes is associated with a more open DNA conformation that, in general, facilitates gene transcription. Conversely, deacetylation of core histones by HDACs is associated with a closed chromatin conformation and (in general) repression of transcription. Therefore, inhibition of HDACs typically leads to derepression of transcription [41,42].

HDACs are the target of a wide number of structurally diverse compounds known as HDAC inhibitors (HDACi) [43]. Accordingly, previous results have demonstrated that VPA is a selective and direct inhibitor of class I HDACs at therapeutic concentrations in humans, causing hyperacetylation of histones in cultured cells by two different mechanisms [32]. On one hand, VPA inhibits the activity of HDACs presumably by binding to the catalytic center of the enzyme. Alternatively, VPA has been shown to reduce HDAC2 protein levels by the ubiquitin−proteasome degradative pathway [41].

Having this in mind, one possibility is that the increased levels of hVDR reporter activity described in this work are due to VPA-mediated inhibition of HDACs, through either direct or indirect mechanisms. This possibility is also supported by the fact that HDACs are recruited by a variety of corepressor complexes and transcription factors, such as Sp1. Indeed, it has been shown that HDAC1 can directly interact with Sp1 via its C-terminal domain [44,45], but as several transcription activators such as E2F1 also interact with the C-terminal domain of Sp1, competition between transcriptional activators and HDACs might be a way to regulate gene expression via reversible chromatin modification [45,46].

There is also increasing evidence of transcription factors being acetylated at lysine residues, thus modifying their transcriptional or binding activity to the DNA response elements [47,48]. Previous studies indicated that Sp1 can be acetylated at Lys703 in the DNA-binding domain, which could alter transcriptional activity, protein−protein interactions, and Sp1-containing protein complexes at the gene promoters [43,49,50]. Although the influence of acetylation and other post-translational modifications on Sp1 is only now becoming apparent, several pieces of evidence demonstrate its impact. Acetylation of Sp1 along with the recruitment of c-Jun transcription factor and p300 increases the activity of the 12(S)-lipoxygenase gene promoter [51]. Moreover, acetylation of Sp1 appears to play a protective role in neuronal cells undergoing oxidative stress via elevated cyclooxygenase 2 (COX-2) expression [52]. It has also been shown that addition of trichostatin A (TSA) enhances acetylation of Sp1 in a time-dependent manner, consistent with the time-dependent increase in TβRII promoter activity [53]. Finally, acetylation of Sp1 and KLF/Sp1 family members, such as EKLF and KLF13, enhances transcriptional potency and affects protein−protein interactions [54,55,56].

In that sense, VPA could inhibit the deacetylation of Sp1 by HDACs—thus disrupting their interaction and reducing the ability to direct HDAC-1 and -2 to target gene promoters. Indeed, a similar mechanism has been described for Npr1 where promoter deletion analysis demonstrated that Sp1 binding sites were essential for HDACi-mediated upregulation of Npr1 promoter activity [43].

From a physiological point of view, VDR activation by SCFAs may lead to different effects. On the one hand, it may have a positive role on cholestatic conditions since it has been shown that active VDR induces CYP3A4 [57,58], which detoxifies toxic hydrophobic bile acids in the liver [59]. On the other, VPA-mediated VDR activation may lead to detrimental effects since we demonstrated that VDR mediates experimental diet-induced liver steatosis [28]. Similarly, gut-specific VDR enhanced weight gain, adipose tissue inflammation, and the development of hepatic steatosis induced by HFD. Altogether, these results demonstrate, that in the liver, VDR can activate and repress lipid metabolism genes that contribute to fat accumulation and to non-alcoholic fatty liver disease (NAFLD) [28]; whereas in the gut, VDR modulates the expression of intestinal factors controlling lipid metabolism in peripheral organs, thus providing a physiological link between VDR signaling in the gut and systemic lipid homeostasis [60]. As elevated hepatic VDR levels have been associated with liver steatosis our results may have clinical relevance regarding hepatic steatosis in patients on VPA therapy, which is not uncommon as 60.9% of VPA-treated patients revealed potential steatosis by ultrasonography [61]. The induction of hVDR by VPA in the liver of these patients could be a potential underlying mechanism.

Finally, the role of VDR in cell-growth inhibition and differentiation in many target tissues [62,63] and the function of VPA as an HDACi upregulating VDR expression, opens the possibility of their use as a potential therapeutic agent for the treatment of malignant diseases. HDACi arrest cell growth and induce differentiation of numerous transformed cell types, including neuroblastoma, erythroleukemia, acute myelogenous leukaemia, and carcinomas of the skin, breast, prostate, bladder, lung, colon, and cervix. HDAC inhibitors have thus been proposed as promising anticancer therapies [64,65,66,67]. However, many HDACi such as TSA do not exhibit isoenzyme selectivity and are of limited therapeutic value due to poor bioavailability in vivo as well as toxic side-effects at high doses [41]. Given the extensive clinical experience with VPA, it may provide a relatively safe, well tested alternative to the use of TSA and trapoxin in the therapy of malignant diseases. Indeed, VPA has been shown to inhibit proliferation and induce differentiation of cell lines derived from human malignant gliomas, and it may well find broader clinical use in the treatment of other types of cancer [68,69,70,71], supporting the use of VPA as an alternative HDACi in the therapy of human malignancies [42,68].

SCFAs, the main products of bacterial fermentation of dietary fibers, have been identified as mediators of diet-induced crosstalk between the microbiome and the host. SCFAs are important for health as they not only provide energy for the intestinal epithelium, but also have many bioactive roles. They regulate immunity, chemotaxis, phagocytosis, and cell proliferation, and have anti-inflammatory, antitumorigenic, and antimicrobial effects, and alter gut integrity [72]. Whether some of these effects are mediated by the transactivation of hVDR by SCFAs remains to be investigated. Our results could also be suggestive of VDR upregulation by SCFAs produced by gut microbiota.

## 5. Conclusions

Altogether, the identification and cloning of this hVDR promoter responsive to VPA and SCFAs provides the basis for future studies aimed at identifying the molecular mechanism of this regulation. Moreover, given the enormous relevance of the VitD3/VDR pathway in human pathophysiology, supported by a wealth of continuous studies, our mechanistic results provide the backdrop and rationale for future translational clinical studies.

## Figures and Tables

**Table 1:** List of primers used in the present work. FW (forward), RV (reverse).

Promoter	Name	Sequence
PBGD-FW	PBGD-FW	CGGAAGAAAACAGCCCAAAGA
PBGD-RV	PBGD-RV	TGAAGCCAGGAGGAAGCACAGT
VDR-FW	VDR-FW	CACCCCTGGGCTCCACTTACC
VDR-RV	VDR-RV	CCGCCACAGGCTGTCCTAGTC
3838-FW	VDR-UP3094-KpnI	CTCGGTACCTCAGTTGTACAATGGAACGGT
3838-RV	VDR-DN6813-HindIII	TGAAAGCTTCTGCCGAAGAGGAGTAAAGG
2514-FW	VDR_UP25869-KpnI	GCTGGTACCGGACTTGGGCAGTAGGAGC
2514-RV	VDR-DN28360-HindIII	GATAAGCTTGGGGAGGGGTGTGCATTATAT
271-FW	508-VDR:161U26	AGCGGTACCGGAGCTCCTGGCAAGAG
271-RV	508-VDR:421L27	ATGCTCGAGGCCGGGCGCTCAGGCCCC
355-FW	508-VDR:433u26	CCCGGTACCGAGCATTAGAGTCTAAG
355-RV	508-VDR:672L27	CAACTCGAGCATCACAGGTGACCATAC
99(1)-FW	VDR-287_1-Sac-UP	AACGGAGCTCCTGGCAAGAGAGGAC
99(1)-RV	VDR-287_1-Hind-DN	TGACAAGCTTAGACAGCCCAGCACC
99(2)-FW	VDR-287_2-Sac-UP	CCAGGAGCTCGGCTGTCTCTGCTTG
99(2)-RV	VDR-287_2-Hind-DN	CCCGAAGCTTCCGGGTTCGCACCTG
63-FW	VDR-287_3-Sac-UP	GTGCGAGCTCGGGAGCAGCGGGAAA
63-RV	VDR-287_3-Hind-DN	GCTCAAGCTTCGGTATCCCAGACGC

## Data Availability

Not applicable.
